# Macrophage Depletion Disrupts Immune Balance and Energy Homeostasis

**DOI:** 10.1371/journal.pone.0099575

**Published:** 2014-06-09

**Authors:** Bonggi Lee, Liping Qiao, Brice Kinney, Gen-Sheng Feng, Jianhua Shao

**Affiliations:** 1 Department of Pediatrics, University of California San Diego, La Jolla, California, United States of America; 2 Department of Pathology, University of California San Diego, La Jolla, California, United States of America; Institut d’Investigacions Biomèdiques August Pi i Sunyer, Spain

## Abstract

Increased macrophage infiltration in tissues including white adipose tissue and skeletal muscle has been recognized as a pro-inflammatory factor that impairs insulin sensitivity in obesity. However, the relationship between tissue macrophages and energy metabolism under non-obese physiological conditions is not clear. To study a homeostatic role of macrophages in energy homeostasis, we depleted tissue macrophages in adult mice through conditional expression of diphtheria toxin (DT) receptor and DT-induced apoptosis. Macrophage depletion robustly reduced body fat mass due to reduced energy intake. These phenotypes were reversed after macrophage recovery. As a potential mechanism, severe hypothalamic and systemic inflammation was induced by neutrophil (NE) infiltration in the absence of macrophages. In addition, macrophage depletion dramatically increased circulating granulocyte colony-stimulating factor (G-CSF) which is indispensable for NE production and tissue infiltration. Our *in vitro* study further revealed that macrophages directly suppress G-CSF gene expression. Therefore, our study indicates that macrophages may play a critical role in integrating immune balance and energy homeostasis under physiological conditions.

## Introduction

Macrophages are resident immune cells found in most of tissues [1]. Recently, their relevance with obesity-induced metabolic syndrome has been highlighted [2]. Increased macrophage infiltration was initially observed in white adipose tissue (WAT) and has been considered a hallmark of obesity-induced tissue inflammation, which deteriorates insulin sensitivity and glucose metabolism [3], [4], [5]. Later studies demonstrated that macrophages infiltrate into other tissues including liver and skeletal muscle in obesity [4], [6], [7], [8]. Therefore, macrophage infiltration has been proposed as a main mechanism of obesity-induced tissue inflammation and insulin resistance.

On the other hand, tissue macrophages are also important in maintaining metabolic homeostasis and tissue structure under non-obese physiological conditions [9]. Macrophages stimulate angiogenesis preceding adipose tissue expansion [10]. Interestingly, weight loss also induces macrophage infiltration into white adipose tissue (WAT), in which the macrophages regulate lipolysis without inducing inflammation [11]. Therefore, macrophages may exhibit different or distinct functions in response to their environmental factors, either maintaining or impairing metabolism. For example, macrophages recruited under conditions of obesity appear to be more inflammatory, which impair insulin signaling in tissues through inducing inflammation [3], [4]. Macrophages present in non-obese conditions appear to be anti-inflammatory, which play a role in maintaining tissue functions [9]. However, recent studies suggest that heterogeneity in the origins of tissue macrophages as well as their polarization makes tissue macrophage population more diverse [12]. Although a certain population of macrophages has been shown to be induced and associated with tissue metabolism under certain pathological circumstances, it is largely unknown whether macrophages modulate systemic energy homeostasis at physiological conditions. In this regard, we depleted tissue macrophages in adult mice and found that macrophages play a role in controlling NE tissue infiltration at least partially by regulating G-CSF production, which was closely associated with alterations in food intake and body composition. Our study suggests homeostatic functions of macrophage to integrate immunity with energy metabolism.

## Materials and Methods

### Mouse Models and Macrophage Depletion

Macrophage-specific diphtheria toxin receptor-expressed mice were used to deplete macrophage. By crossing inducible diphtheria toxin receptor mice (iDTR, C57BL/6 background, Jackson Laboratory) with lysozyme M promoter-directed CRE mice (LysM^Cre^, C57BL/6 background, Jackson Laboratory), LysM^Cre^/iDTR mice were generated to express Cre recombinase in macrophages where Cre excised the STOP cassette and led to DTR expression [13], [14], [15]. Male mice (2–3 months old) were used for this study and fed either a standard chow (Harlan Laboratories) or 60% HF diet for 6 wks (Research Diet). Diphtheria toxin (DT) was injected intraperitoneally every other day (10****ng/g body weight). If it is not mentioned in the figure legends, tissue samples were collected at day 6 of DT treatment. All mice were maintained under standardized conditions with 12 h/12 h light/dark cycles. Mice were sacrificed by CO_2_ inhalation. The experiments using mouse models were carried out under the Association for Assessment and Accreditation of Laboratory Animal Care guidelines with approval of the University of California San Diego Animal Care and Use Committee (Animal protocol number S09123).

### Body Weight, Composition and Indirect Calorimetry Assay

Body weight and composition were measured using a mouse MRI scanning device (EchoMRI system) when mice were at fed state and these masses were calculated as a percent change compared to day 0 of saline or DT injection. Oxygen consumption is assessed by an indirect calorimetric system (Oxymax, Columbus Instruments). Mice were acclimated to the system for 12 hrs and data were collected every 8****minutes for 24****hrs under light/dark cycles with free access to water and food.

### Serum Cytokine Assay and Blood Cells Count

Serum concentrations of cytokines were measured using mouse cytokine array/chemokine array 32-plex panel (Bio-Rad) through a service by the Eve Technologies (Calgary, Alberta). For measuring NE concentration in blood, a hematology test was performed by University of California San Diego Murine Hematology and Coagulation Core Laboratory.

### Microglia and Peritoneal Macrophage Culture

The method for microglia isolation was modified from a previous study [16]. Brains isolated from newborn mice were dissociated by nylon mesh (75 micron mesh) in chilled Hanks’ balanced salt solution (HBSS) and washed 2 times with HBSS. The cell suspension was triturated with a glass pipette and plated in a F75 flask in Dulbecco’s Modified Eagle’s Medium (DMEM) supplemented with 10% FBS. Medium was changed every 3 days. On the 14th day, microglia were isolated by mechanical agitation (150 rpm for 2 hrs) in an orbital shaker at 37°C. The isolated cells were plated again in a non-coated plastic dish for 30 mins. Non-attached cells were removed by washing with DMEM. Attached microglia were used for further experiments.

Peritoneal macrophages were induced by thioglycollate broth. Briefly, peritoneal fluid was collected 3 days after thioglycollate broth injection (3****ml per mouse) and macrophages were cultured in 10****cm tissue culture plates using DMEM supplemented with 10% FBS [17]. Cultured macrophages were treated with saline or DT (0.25****ng/ml) for further experiments.

### Immunoblots and Immunohistochemistry

For immunoblotting, mechanically homogenized tissue samples were separated using NuPAGE gels (Invitrogen). Protein was blotted with indicated antibodies. Protein levels were semiquantified by image density scanning using Quantity One software (Bio-Rad) with an internal reference.

Immunohistochemistry analysis was carried out by the Histology Core at UCSD. Mice were perfused using 4% paraformaldehyde and then each tissue was removed, fixed in 10% formaldehyde, embedded in paraffin and then sectioned. Tissue layers were labeled with NE marker (NIMP-R14, LY-6G) or microglia marker (Iba1), or used for H&E staining.

The antibody labeled tissue layers were visualized by 3-amino-9-ethylcarbazole (AEC) substrate kit (Cat # SK4200, Vector laboratories).

### Quantitative PCR Analysis

Total RNA was prepared from homogenized epididymal fat, liver, skeletal muscle (quadriceps), and hypothalamus with Trizol (Invitrogen). cDNA was synthesized using SuperScript III Reverse Transcriptase and oligo(dT)12–18 primer (Invitrogen). Real-time PCR was performed using an mx3000p Real-Time PCR system (Stratagene) and SYBR Green dye (Molecular Probes).

### OP9 Bone-marrow Stromal Cell Culture

OP9 cells were grown to 100% confluence in minimum essential media (MEM) supplemented with 10% FBS in 6 well plates. OP9 cells were cultured with either MEM or MEM including peritoneal or bone-marrow macrophage conditioned medium (10%) with lipopolysaccharide stimulation to induce G-CSF expression. After overnight incubation, the cells were treated with Trizol for RNA extraction.

### Statistical Analysis

Data are expressed as mean ± standard error of the mean (SEM). The Prism software (GraphPad Software, Inc.) was used for statistical analysis. The Student’s t-tests (two-tailed) were used for pairwise comparisons. Differences were considered significant when p<0.05.

## Results

### Macrophage Depletion Decreases Body Weight Including Fat and Lean Mass

**Figure 1:**
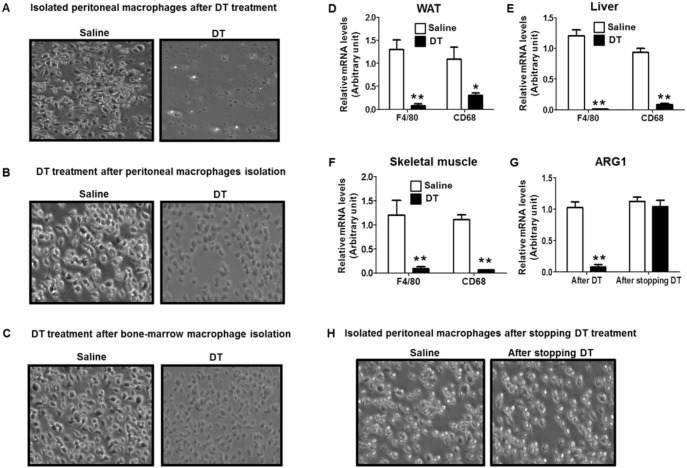
DT treatment efficiently depletes macrophages. For microscopic images of macrophages, macrophages were collected from peritoneal lavage of LysM^Cre^/iDTR mice (A) 2 days after DT treatment (10 ng/g body weight) and (H) 7 days after stopping DT injection. Macrophages were collected from (B) peritoneal lavage or (C) bone-marrow of LysM^Cre^/iDTR mice, then treated with saline or DT (0.25 ng/ml) for 8 hours. mRNA levels of macrophage marker F4/80 and CD68 were measured in (D) WAT, (E) liver, and (F) skeletal muscle of mice treated with saline or DT for 2 days. mRNA levels of M2 macrophage marker ARG1 were measured in (G) WAT of mice treated with saline or DT for 2 days and 7 days after stopping DT injection. Data are represented as mean ± SEM. Asterisks denote significant differences *P<0.05, **P<0.01 vs. saline-treated mice.

To investigate the physiological function of macrophages in systemic energy metabolism, we depleted macrophages in adult mice using Lysozyme M-directed myeloid-specific diphtheria toxin receptor transgenic mice (LysM^Cre^/iDTR) [13]. Following DT administration, ectopically expressed DT receptors promptly induced macrophage apoptosis [14], [15] (Figure 1A–C). In addition, general macrophage markers such as F4/80 and CD68 were markedly reduced in skeletal muscle, WAT, and liver of DT-treated mice (Figure 1D–F). M2 macrophage marker ARG1 was also robustly reduced in WAT of DT-treated mice (Figure 1G). These results indicate that DT injection induced macrophage depletion regardless of its activation state in LysM^Cre^/iDTR mice. DT injection did not induce fever, which is a sign of bacterial infection in LysM^Cre^/iDTR mice (data not shown).

**Figure 2:**
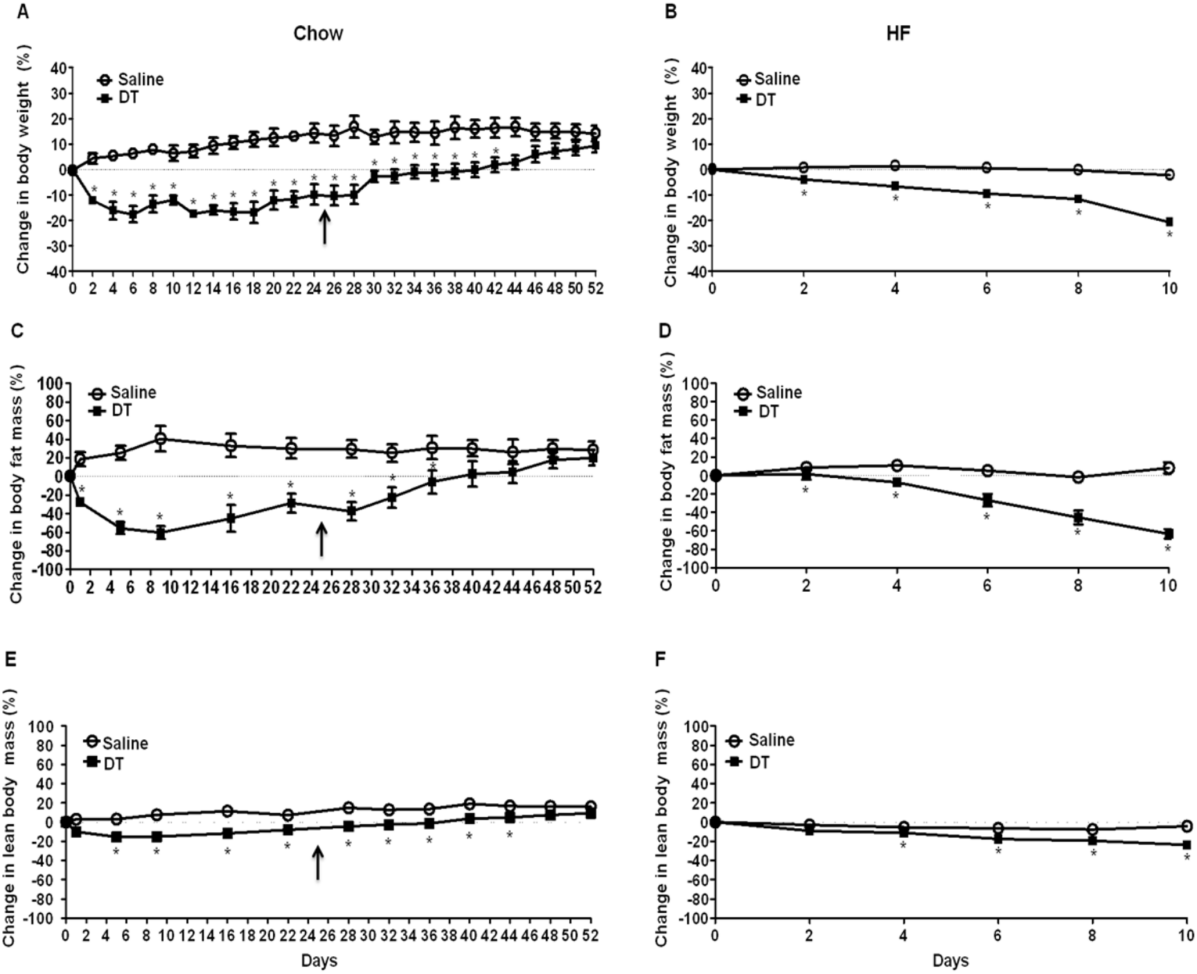
Macrophage depletion reduces body weight and fat mass. Male LysM^Cre^/iDTR mice were fed (A, C, and E) a chow diet (n = 7) or (B, D, and F) a HF diet (n = 5) for 6 wks and i.p. injected with DT (10 ng/g) or saline every other day. For chow-fed mice, DT injection was stopped at day 25 which is indicated by arrows. (A and B) Body weight was measured every other day and calculated as a percent change compared to day 0 of saline or DT injection (C and D) Fat mass and (E and F) lean body mass were measured using an EchoMRI scanning device when mice were at fed state and these masses were calculated as a percent change compared to day 0 of saline or DT injection. Data are presented as mean ± SEM. *P<0.05 vs. saline-treated mice.

DT treatment induced a robust reduction in body weight with a significant decrease in fat mass and lean tissue mass in chow-fed and HF-fed LysM^Cre^/iDTR mice (Figure 2A–F). The reduction of fat tissue mass reached its maximal point (∼60%) 9 days after DT treatment (Figure 2C). Conversely, approximately 7 days after stopping DT treatment, mice started to recover fat mass and lean body mass (Figure 2C and E). During the same time, tissue macrophage marker expression (data not shown) and the yield of thioglycollate broth-induced peritoneal macrophages were recovered (Figure 1H). However, DT injection showed no effect on body weight or food intake in C57BL/6 mice and mice without Cre recombinase insertion (LysM^Cre−/−^/iDTR) (data not shown), suggesting that DT does not have any effects on energy metabolism in mice without DT receptors. These results suggest that DT-induced reduction in body weight and fat tissue mass is closely associated with macrophage ablation.

### Macrophage Depletion Reduces Food Intake and STAT3 Signaling in the Hypothalamus

**Figure 3:**
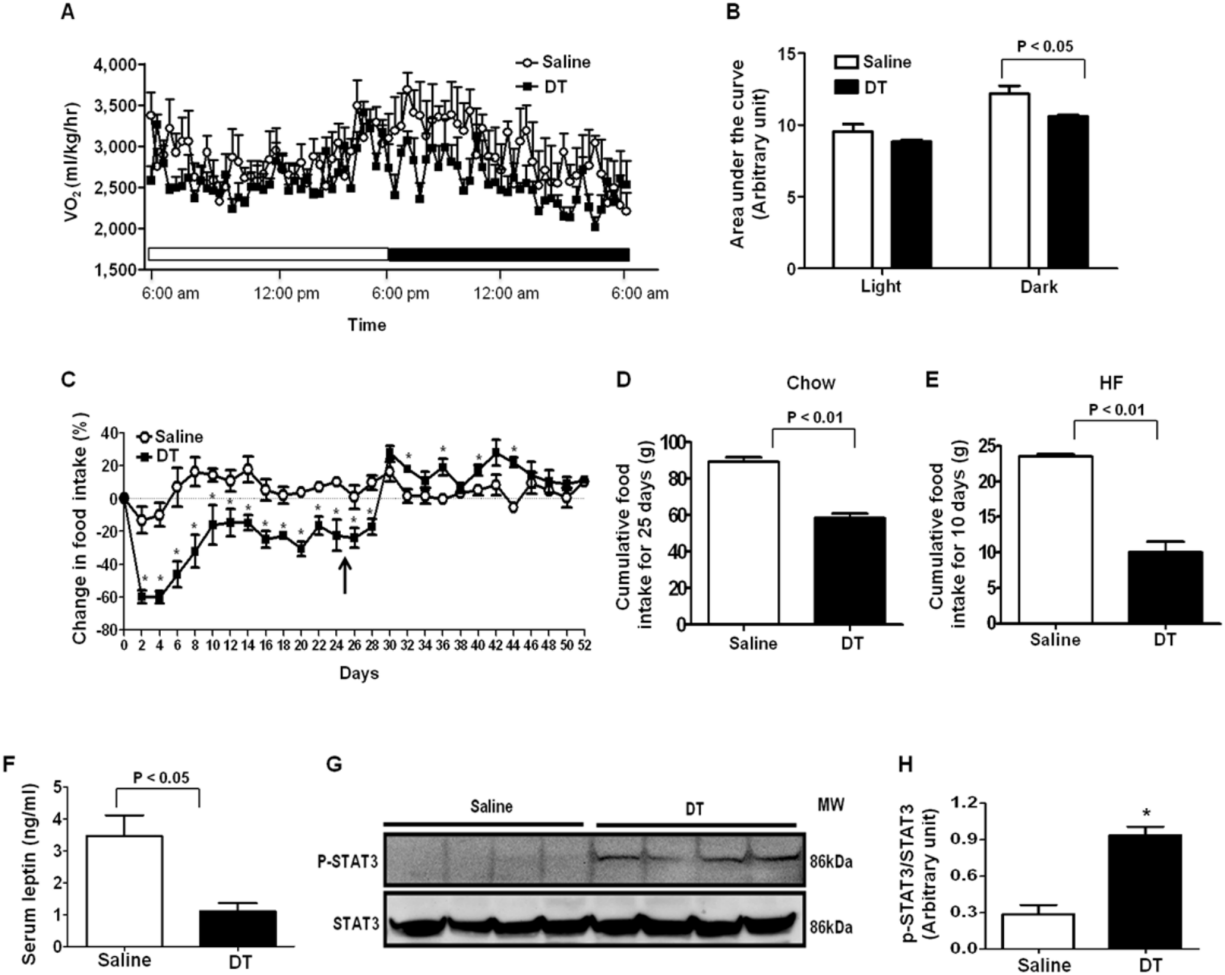
Macrophage depletion suppresses energy expenditure and food intake. The energy expenditure of male LysM^Cre^/iDTR mice was assessed using metabolic cages 6 days after saline or DT injection. (A) O_2_ consumption during the light and dark cycle (n = 4/each group) (B) Area under the curve of O2 consumption during the light and dark cycle (C) Food intake was measured every other day at 10 am during saline or DT injection. Injection was stopped at day 25, which is indicated by arrows. Cumulative food intake of (D) chow-fed (n = 5/group) or (E) HF-fed mice (n = 3/group) treated with saline or DT (F) Serum concentration of leptin (n = 5/group) was measured using ELISA kit after overnight fasting. (G) Phosphorylation of STAT3 protein was measured by Western blotting. (H) Quantified values of p-STAT3/STAT3. Data are represented as mean ± SEM. Asterisks denote significant differences *P<0.05 vs. saline-treated mice.

Under physiological conditions, energy balance determines fat tissue mass. We measured food intake and energy expenditure to verify which of these factors contributes to body fat decrease in macrophage-depleted mice. Although there were no significant differences in energy expenditure between groups during the light cycle (Figure 3A–B), reduced energy expenditure was observed in DT-treated mice during the dark cycle (Figure 3A–B). Impressively, just after a single DT injection, a remarkable decrease in food intake was observed in DT-treated mice (Figure 3C). Although there was a partial recovery of food intake after prolonged DT treatment (Figure 3C), food intake was significantly lower during the DT injection period (Figure 3C). The cumulative food intake of DT-injected mice was markedly reduced in both chow and HF-fed conditions (Figure 3D–E). After stopping DT treatment, food intake was recovered (Figure 3C), which paralleled the body weight recovery (Figure 2A). Together, these data strongly indicate that reduced food intake is the main cause of the decrease in body weight in macrophage-depleted mice.

Leptin is an adipocyte-derived satiety hormone that inhibits food intake through neurons in the hypothalamus [18]. Our results showed that there was a significant decrease in serum leptin levels in DT-treated LysM^Cre^/iDTR mice (Figure 3F). Because DT-treated LysM^Cre^/iDTR mice ate less than control mice, reduction in circulating leptin should not be responsible for macrophage depletion-induced reduction in food intake and fat tissue mass. We studied a signaling pathway in the hypothalamus that controls food intake. STAT3 is a transcription factor which is known for mediating the regulatory effects of hormones including leptin and inflammatory cytokines on food intake. Phosphorylation of STAT3 in the hypothalamus leads to reduction in food intake [18], [19]. Our results showed that the STAT3 phosphorylation levels were robustly elevated in the hypothalamus of DT-treated LysM^Cre^/iDTR mice (Figure 3G–H). Therefore, the increased STAT3 phosphorylation in the hypothalamus and the decreased food intake suggest that macrophage ablation suppresses food intake through a mechanism(s) that increases STAT3 phosphorylation in the hypothalamus.

### DT Treatment Ablates Microglia in Culture but not in LysM^Cre^/iDTR Mice

**Figure 4:**
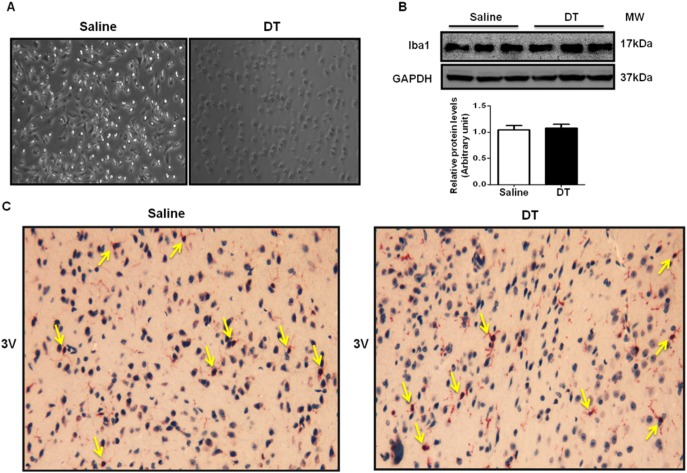
DT depletes microglia in Microglia were isolated from day 1 newborn LysM^Cre^/iDTR mice (n = 3). Isolated microglia were cultured in cell-culture plates. Saline or DT (0.25 ng/ml) was treated for 8 hrs. (A) Representative macroscopic images of isolated microglia treated with saline or DT for 8 hrs. (B) Total protein levels of microglia marker Iba1 in the hypothalamus of mice treated with saline or DT for 6 days. (C) Microglia in the brain were identified by immunohistochemisty using microglia marker Iba1 antibody. Arrows indicate positive cells (n = 3). 3 V represents the direction of third ventricle.

Microglia are macrophages in the CNS, which raises a question of whether DT treatment induces microglia apoptosis. Using cultured primary microglia from LysM^Cre^/iDTR mice, our study showed that DT treatment killed all the cells (Figure 4A), suggesting that Lysozyme M promoter-derived DT receptor was expressed in microglia. However, intraperitoneal injection of DT into mice did not alter microglia population in the brain, evidenced by similar levels of microglia-specific marker Iba1 in the hypothalamus (Figure 4B). Immunohistochemisty staining further confirmed the similar distribution of Iba1 in the hypothalamus of DT-treated LysM^Cre^/iDTR and control mice (Figure 4C), indicating that *in vivo* microglia evaded DT-induced apoptosis. However, by using the similar system, a study has reported that DT injection depleted oligodendrocytes in the brain [13], suggesting that DT is able to go through the blood-brain barrier. The DT dosage used in this study is approximately 2–3 times higher than our study. Therefore, we postulate that the low dosage of DT used in our study may elude microglia from DT induced apoptosis in LysM^Cre^/iDTR mice.

### Macrophage Depletion Induces Hypothalamic ER Stress and Inflammation

**Figure 5:**
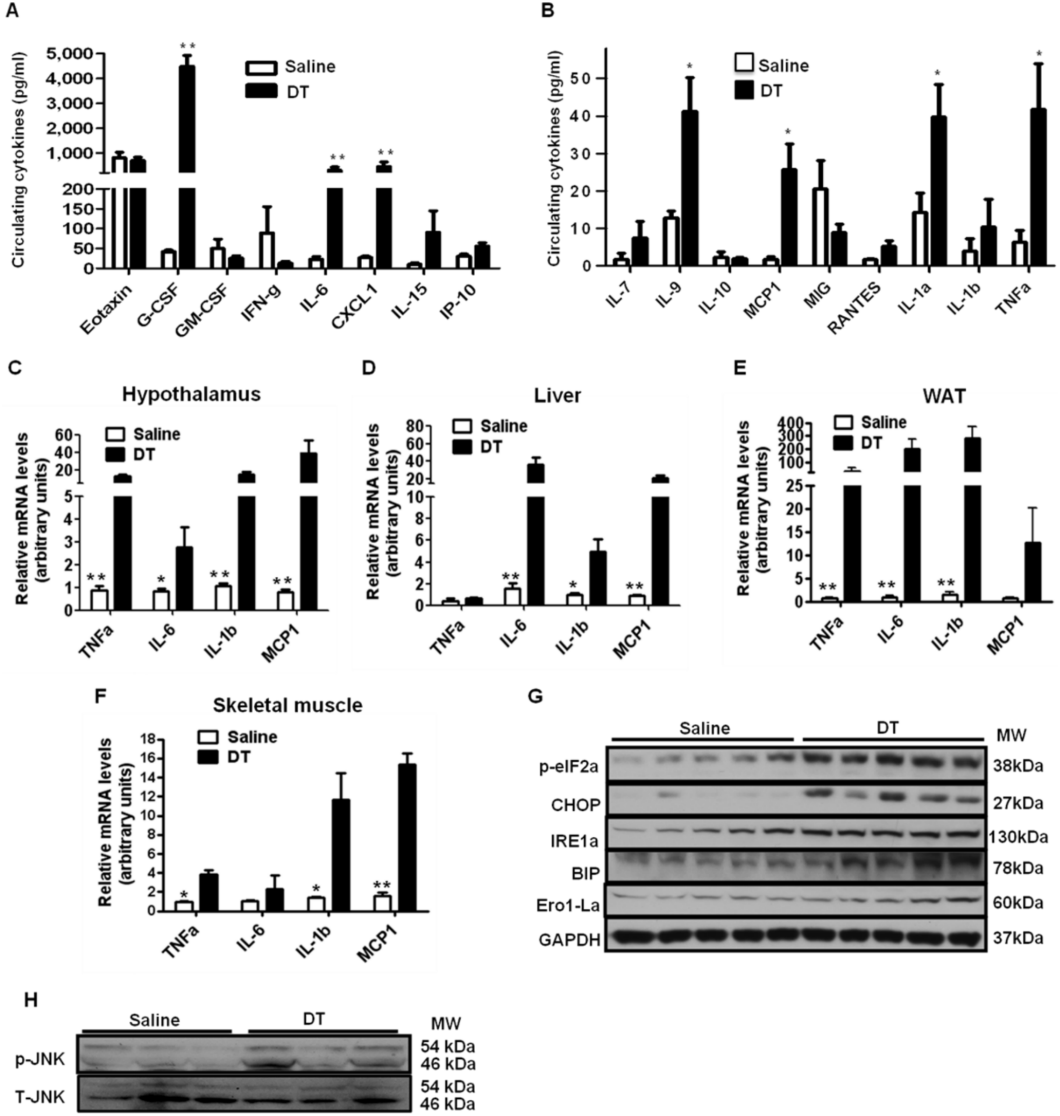
Macrophage depletion increases hypothalamic and systemic inflammation. Male LysM^Cre^/iDTR mice were i.p. injected with DT (10 ng/g) or saline for 6 days. Blood and tissue samples were collected after overnight fasting. (A and B) Serum concentrations of cytokines were measured using a mouse cytokine/chemokine array service by a commercial company (n = 8–10/group). (C–F) mRNA levels of pro-inflammatory cytokine expression in the hypothalamus, liver, WAT, and skeletal muscle of saline or DT treated mice (n = 5 each group). (G) Protein levels related to ER-stress signaling and (H) phosphorylation of JNK were measured by Western blotting. G-CSF, granulocyte colony-stimulating factor; GM-CSF, granulocyte macrophage colony-stimulating factor; CXCL, C-X-C motif chemokine; IL-6, interleukin-6; TNFα, tumor necrosis factor α; MCP1, monocyte chemotactic protein-1; MIG, monokine-induced by Interferon-gamma; RANTES, Chemokine (C-C motif) ligand 5. Data are represented as mean ± SEM. Asterisks denote significant differences *P<0.05, **P<0.01 vs. saline-treated mice.

Food intake can be inhibited by hypothalamic inflammation which is usually induced by local or systemic factors such as circulating inflammatory cytokines [20], [21]. Peripheral inflammatory conditions have been found to elevate expression and release of inflammatory cytokines within the CNS [21]. Elevated CNS inflammatory cytokines have been known to decrease food intake by stimulating STAT3 and its downstream signals [19], [21], [22], [23], [24]. To examine a potential factor that contributes to the reduced food intake after macrophage depletion, we measured circulating pro-inflammatory cytokines as well as the extent of inflammation in the hypothalamus. The levels of circulating pro-inflammatory cytokines, such as TNFα (∼7 fold), IL-6 (∼13 fold), IL-7 (∼4 fold), IL-9 (∼3 fold), CXCL1 (∼17 fold) and MCP1 (∼15 fold), were dramatically increased in DT-treated mice (Figure 5A–B) and reversed after macrophage recovery by stopping DT treatment (data not shown). Furthermore, the expression levels of pro-inflammatory cytokines, such as TNFα, IL-6, IL-1β, and MCP1, were markedly increased in the hypothalamus of DT-treated mice (Figure 5C). When the increase in the inflammatory gene expression was reversed in the hypothalamus after stopping DT injection (data not shown), food intake was also recovered (Figure 3C), indicating close association between the hypothalamic inflammation and decreased food intake.

JNK signaling and ER stress are known as key factors related to hypothalamic inflammatory and stress responses [21], [25]. To examine whether local inflammatory pathways are also involved in the increased hypothalamic inflammation, we measured JNK and ER stress signaling. Immunoblotting data shows that the levels of ER stress-related proteins including phosphorylated eIF2a, CHOP, IRE1a, BIP, and Ero1-La as well as phosphorylated JNK, were markedly increased in the hypothalamus of DT-treated mice (Figure 5G–H). These results suggest that local inflammatory pathways are also involved in the increased hypothalamic inflammation. Therefore, both systemic and local factors are associated with increased hypothalamic inflammation after macrophage depletion.

### Macrophage Depletion Induces Inflammation in Peripheral Tissues

Macrophages are known for secreting pro-inflammatory cytokines and inducing tissue inflammatory reactions [4]. The dramatically increased blood inflammatory cytokines in DT-treated LysM^Cre^/iDTR mice led us to explore the origin of the cytokines in the absence of macrophage. We compared gene expression levels of pro-inflammatory cytokines in peripheral tissues between DT- and saline-treated LysM^Cre^/iDTR mice. Significantly increased mRNA levels of TNFα, IL-6, IL-1β and MCP1 were observed in the liver, skeletal muscle, and more markedly in WAT of DT-treated LysM^Cre^/iDTR mice (Figure 5D–F), suggesting that macrophage depletion elevates pro-inflammatory cytokine expression in peripheral tissues, which may contribute to the increase in blood pro-inflammatory cytokine levels.

### Neutrophils Infiltration Occurs in Tissues in the Absence of Macrophages

**Figure 6:**
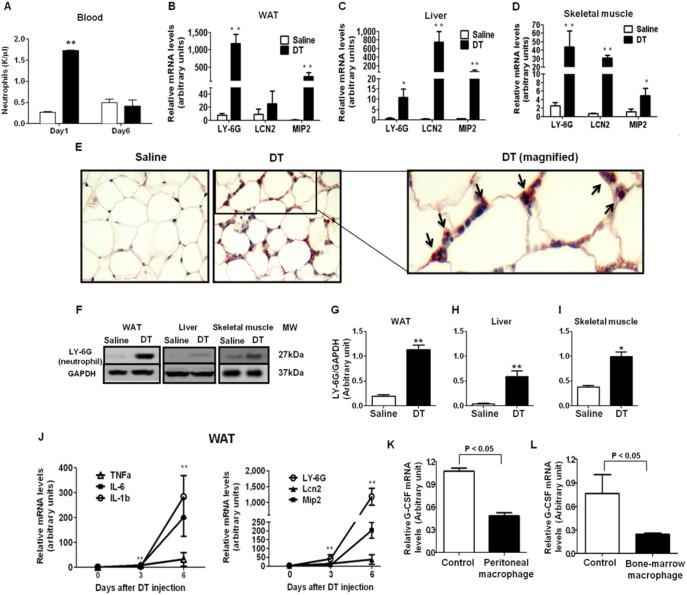
Macrophage depletion increases tissue NE infiltration. Male LysM^Cre^/iDTR mice were fed a chow diet and were i.p. injected with DT (10 ng/g) or saline. (A) Blood NE concentration was assessed after 1 and 6 days of DT injection by a hematology test. (B–D) mRNA levels of NE markers LY-6G, LCN, and NE-chemoattractant chemokine MIP2 in WAT, liver, and skeletal muscle were measured by Q-PCR (n = 5). LY-6G, lymphocyte antigen 6 complex, locus G; MIP2, macrophage inflammatory protein 2; LCN2, lipocalin 2 (E) Infiltrated NEs in WAT were identified by immunohistochemisty using NE marker LY-6G antibody. Arrows indicate positive cells. (F) Protein levels of NE marker LY-6G in WAT, liver, and skeletal muscle were measured by Western blotting (n = 4 each). Representative images were shown in the figure. (G–I) Quantification of LY-6G/GAPDH protein levels in tissues. (J) The comparison of inflammatory cytokine expression with NE marker expression in WAT by Q-PCR during the different time points of DT treatment. (K and L) G-CSF mRNA levels were measured by Q-PCR using RNA samples from OP9 cells treated with 10% peritoneal (K) or bone-marrow derived macrophage (L) conditioned media (n = 4 each). Data are represented as mean ± SEM. Asterisks denote significant differences *P<0.05, **P<0.01 vs. saline-treated mice.

Neutrophils (NEs) are the most abundant immune cells in the blood that produce a wide range of pro-inflammatory cytokines and chemokines [26], [27]. NEs are also known for acting together with macrophages in the process of immune response and wound healing [14], [15], [28]. There was an increase in NE infiltration in wound tissues after macrophage depletion [14], [15], [28]. In addition, macrophage depletion increases NE in circulation in physiological conditions [29]. Therefore, we investigated whether macrophage depletion increases NE in circulation and tissues and whether NEs are involved in macrophage depletion-induced increases in pro-inflammatory cytokines in circulation and in tissues. Hematology test showed that blood NE counts were significantly increased 1 day after DT injection but recovered to the level comparable to the control group 6 days after DT injection (Figure 6A). Furthermore, mRNA expression levels of NE-specific markers (LY-6G and LCN2) were dramatically increased in WAT, skeletal muscle, and liver of DT-treated LysM^Cre^/iDTR mice (Figure 6B–D). Similarly, immunoblotting showed an increase in LY-6G protein levels in these tissues (Figure 6F–I). Histology staining further confirmed the infiltration of NEs into WAT (Figure 6E). On the other hand, immunohistochemisty using NE marker (LY-6G) showed that NEs were not infiltrated in the hypothalamus of both groups (data not shown).

MIP2 (also known as CXCL2) is a prototypical NE attracting chemokine [14]. It has been shown that macrophage depletion highly increased the mRNA expression of MIP2 in a wound tissue [14]. We examined whether macrophage depletion alters MIP2 expression in tissues and found the mRNA expression of MIP2 was notably increased in WAT, skeletal muscle, and liver of DT-treated LysM^Cre^/iDTR mice (Figure 6B–D), indicating that the increased MIP2 expression likely contributes to NE infiltration into these tissues.

NEs are considered a hallmark of inflammation [26], [27]. We compared the expression levels of pro-inflammatory cytokine with NE marker in WAT at 0, 3, and 6 days of DT injection. The pattern of pro-inflammatory cytokine expression was similar to the pattern of NE marker expression (Figure 6J), suggesting that NE infiltration is closely associated with the development of tissue inflammation.

### Macrophage-secreted Factors Directly Inhibit G-CSF Expression

To examine why macrophage depletion leads to dramatic NE infiltration into tissues, we measured serum concentration of granulocyte colony-stimulating factor (G-CSF) which is essential for NE production and tissue infiltration [29], [30], [31], [32]. Our results showed that there was a 110-fold increase in serum G-CSF levels of DT-treated mice (Figure 5A) and the increased G-CSF levels were reversed after macrophage recovery (data not shown). These results suggest that increased G-CSF is closely related to macrophage depletion-induced NE infiltration. Similarly, a recent mouse study reported a marked increase in circulating G-CSF after macrophage depletion in bone-marrow and spleen, which was associated with NE infiltration into macrophage-depleted tissues [29]. Therefore, we hypothesized that macrophages directly regulate G-CSF expression. We used OP9 bone-marrow stromal cells, which have been known to secrete G-CSF especially after stimulation by either lipopolysaccharide or IL-1 [33], [34], [35]. To study whether macrophages regulate G-CSF expression through secreted cytokines, we treated OP9 cells with conditioned media from either bone-marrow or peritoneal derived macrophages of wild type mice. The results showed that G-CSF mRNA levels were significantly reduced (>50%) in macrophage conditioned media-treated OP9 cells (Figure 6K–L), suggesting that macrophages directly suppress G-CSF gene expression.

## Discussion

The studies related to obesity-induced tissue macrophage infiltration have demonstrated a close association of energy metabolism with immunity [3], [4]. Although these studies indicate that prolonged positive energy imbalance induces pro-inflammatory macrophages infiltration, it is largely unknown whether immune balance actively contributes to energy homeostasis. With development of conditional gene knockout mouse models, recent studies have provided evidence suggesting that immunity plays an active role in energy metabolism. For example, macrophage-specific PPARγ deficiency impaired M2 macrophage polarization and predisposed the mice to HF diet-induced obesity [36]. PPARδ deficiency impaired M2 activation of kupffer cells, which was related to increased fat mass in HF-fed mice [37]. However, a heterogeneous population of macrophages exists under non-obese conditions and their role in regulating energy metabolism has not been studied [9]. By depleting tissue macrophages, our study revealed that macrophage depletion notably reduced body weight including fat and lean mass by inhibiting food intake. This study demonstrates that macrophages are involved in maintaining energy homeostasis in physiological conditions. In addition, our study showed that macrophage depletion robustly increased pro-inflammatory cytokines expression and their levels in circulation, suggesting that macrophage depletion disturbs immune balance. Therefore, we propose that there may be reciprocal regulation between immune balance and energy metabolism.

Hypothalamic inflammation can either increase or decrease food intake probably depending on the state of inflammation [21], [25]. Relatively mild and chronic inflammation related to diet-induced obesity has been shown to inhibit leptin signaling and thereby increasing food intake [25]. On the other hand, relatively acute and robust hypothalamic inflammation achieved by inflammatory cytokine injection decrease food intake by stimulating STAT3 signaling pathway [19], [21], [22], [23], [24]. Our results showed that macrophage depletion notably increased inflammatory cytokine expression (Figure 5C), JNK and ER stress signaling (Figure 5G–H), and STAT3 phosphorylation in the hypothalamus (Figure 3G–H). Considering notable reduction in circulating leptin levels and food intake in chow-fed lean mice after DT injection (Figure 3F), we speculate that markedly increased inflammatory cytokine in circulation (Figure 5A–B) may cause robust hypothalamic inflammation independent of obesity-related leptin resistance. This may contribute to reduced food intake in the absence of macrophages. These results indicate that macrophages are involved in controlling food intake at least partially by maintaining hypothalamic immune balance. Since microglia, macrophages in CNS, were evaded from DT-induced apoptosis, it is likely that hypothalamic immune balance was impaired by circulating factors in the absence of macrophages. In support of this, it has been reported that the circulating inflammatory cytokines can induce hypothalamic inflammation and reduction in food intake [21], [22]. We also found that there were dramatic increases in pro-inflammatory cytokines in blood (Figure 5A–B), which has been known to induce CNS inflammation and reduce food intake.

Short-lived NEs are generally considered the first immune cell to respond to inflammation [38]. The timely clearance of apoptotic NEs from inflammation sites is an important function of macrophages [27], [29]. Therefore, ablation of wound macrophages increased NE recruitment and inflammation at the site of injury [14], [15]. However, the role of macrophages in regulating NE infiltration has not been fully understood in intact tissues. Our study shows that macrophage depletion notably increases NE infiltration and inflammatory cytokine expression in tissues (Figure 6B–I) indicating that macrophages may not only regulate NE infiltration during wound healing process, but also control them in intact tissues in physiological conditions. NEs induce inflammation through secreting a wide range of inflammatory cytokines [26]. Also, NEs infiltration itself can be considered as a marker of inflammation [26], [27]. Our study also indicates that increased inflammation is closely associated with increased NE infiltration in adipose tissue (Figure 6j). Therefore, we speculate that increased tissue inflammation in the absence of macrophages may be caused by infiltrated NEs. G-CSF is an essential hormone to stimulate NE production and tissue infiltration [29], [30], [31], [32]. Our data showed that tissue macrophage depletion dramatically increased G-CSF in circulation (Figure 5A) and NE infiltration into tissues (Figure 6B–I). Therefore, our data suggest that macrophages may play a role in maintaining tissue immune balance by regulating circulating G-CSF and NE infiltration.

NEs also express lysozyme M [14], [15]. Therefore, it raises a question whether DT treatment also deplete NEs in LysM^Cre^/iDTR mice. Our study showed that blood NEs were notably increased 1 day after DT treatment and returned to the levels comparable to the control group (Figure 6A). NE marker expression was even dramatically increased in adipose tissue 6 days after DT treatment (Figure 6J). These results argue against the notion that DT also induces NE apoptosis in LysM^Cre^/iDTR mice. Although more studies are required to directly verify this notion, previous studies using the same mouse model also reported the similar effects on NEs when even higher dosage of DT was injected [14], [15], [39]. One study indicates that analysis of adherent, differentiated primary macrophages (CD11b and F4/80 high) and freshly isolated neutrophils (Ly6G high) showed notably reduced lysozyme M transcript levels in neutrophils [14]. In addition, DT also specifically ablated a CD11b high and LY6C low immune cell population (macrophages) from thioglycollate-induced peritoneal infiltrate in LysM^Cre^/iDTR mice [14]. Therefore, it is likely that compared to macrophages, NEs are less sensitive to DT-induced apoptosis at least partially due to low lysozyme M promoter activity in these cells [14], [15], [39].

Since phagocytosis is a main function of macrophages for removal of cellular debris, it is unclear whether DT-induced macrophage apoptosis causes the tissue inflammation and elevation of blood pro-inflammatory cytokines. However, a study using iDTR mouse model to delete CD-11C positive cells showed no sign of severe inflammation and have no effect on body weight [8]. Another study partially depleted splenic marginal zone macrophages and ∼70% of bone marrow stromal macrophages by conditionally deleting the anti-apoptotic gene, FLICE-like inhibitory protein in macrophages using LysM^Cre^ mice [29]. Similar to our observation, they found that although macrophages were partially depleted and the dead macrophages could be removed by surviving macrophages, these mice develop severe neutrophilia with decreased body weight and increased of G-CSF and inflammatory cytokines [29]. In addition, our *in vitro* study showed that macrophage-conditioned medium directly reduced the gene expression of G-CSF that plays an important role in NE production and tissue infiltration (Figure 6J–K). Together, these data lead us to hypothesize that macrophages may directly regulate NEs activation and tissue infiltration. It may underlie macrophage depletion-induced inflammation and alteration in energy metabolism. However, further studies are necessary to verify this notion and to elucidate whether NE infiltration is secondary to apoptotic macrophages-induced inflammation.

In summary, our study suggests that macrophage plays a key role in maintaining immune balance and energy homeostasis. In the absence of macrophages there is an up-surge of G-CSF followed by NE infiltration into tissues, which induces tissue inflammation and body weight reduction due to decreased food intake.

## References

[1] Gordon S, Taylor PR (2005) Monocyte and macrophage heterogeneity. Nat Rev Immunol 5: 953–964. 16322748 10.1038/nri1733

[2] Hotamisligil GS (2006) Inflammation and metabolic disorders. Nature 444: 860–867. 17167474 10.1038/nature05485

[3] Odegaard JI, Chawla A (2008) Mechanisms of macrophage activation in obesity-induced insulin resistance. Nat Clin Pract Endocrinol Metab 4: 619–626. 18838972 10.1038/ncpendmet0976 PMC3381907

[4] Olefsky JM, Glass CK (2010) Macrophages, inflammation, and insulin resistance. Annu Rev Physiol 72: 219–246. 20148674 10.1146/annurev-physiol-021909-135846

[5] Weisberg SP, McCann D, Desai M, Rosenbaum M, Leibel RL, et al (2003) Obesity is associated with macrophage accumulation in adipose tissue. J Clin Invest 112: 1796–1808. 14679176 10.1172/JCI19246 PMC296995

[6] Arkan MC, Hevener AL, Greten FR, Maeda S, Li ZW, et al (2005) IKK-beta links inflammation to obesity-induced insulin resistance. Nat Med 11: 191–198. 15685170 10.1038/nm1185

[7] Nguyen MT, Favelyukis S, Nguyen AK, Reichart D, Scott PA, et al (2007) A subpopulation of macrophages infiltrates hypertrophic adipose tissue and is activated by free fatty acids via Toll-like receptors 2 and 4 and JNK-dependent pathways. J Biol Chem 282: 35279–35292. 17916553 10.1074/jbc.M706762200

[8] Patsouris D, Li PP, Thapar D, Chapman J, Olefsky JM, et al (2008) Ablation of CD11c-positive cells normalizes insulin sensitivity in obese insulin resistant animals. Cell Metab 8: 301–309. 18840360 10.1016/j.cmet.2008.08.015 PMC2630775

[9] Odegaard JI, Chawla A (2011) Alternative macrophage activation and metabolism. Annu Rev Pathol 6: 275–297. 21034223 10.1146/annurev-pathol-011110-130138 PMC3381938

[10] Pang C, Gao Z, Yin J, Zhang J, Jia W, et al (2008) Macrophage infiltration into adipose tissue may promote angiogenesis for adipose tissue remodeling in obesity. Am J Physiol Endocrinol Metab 295: E313–322. 18492768 10.1152/ajpendo.90296.2008 PMC2519760

[11] Kosteli A, Sugaru E, Haemmerle G, Martin JF, Lei J, et al (2010) Weight loss and lipolysis promote a dynamic immune response in murine adipose tissue. J Clin Invest 120: 3466–3479. 20877011 10.1172/JCI42845 PMC2947229

[12] Davies LC, Jenkins SJ, Allen JE, Taylor PR (2013) Tissue-resident macrophages. Nat Immunol 14: 986–995. 24048120 10.1038/ni.2705 PMC4045180

[13] Buch T, Heppner FL, Tertilt C, Heinen TJ, Kremer M, et al (2005) A Cre-inducible diphtheria toxin receptor mediates cell lineage ablation after toxin administration. Nat Methods 2: 419–426. 15908920 10.1038/nmeth762

[14] Goren I, Allmann N, Yogev N, Schurmann C, Linke A, et al (2009) A transgenic mouse model of inducible macrophage depletion: effects of diphtheria toxin-driven lysozyme M-specific cell lineage ablation on wound inflammatory, angiogenic, and contractive processes. Am J Pathol 175: 132–147. 19528348 10.2353/ajpath.2009.081002 PMC2708801

[15] Lucas T, Waisman A, Ranjan R, Roes J, Krieg T, et al (2010) Differential Roles of Macrophages in Diverse Phases of Skin Repair. The Journal of Immunology 184: 3964–3977. 20176743 10.4049/jimmunol.0903356

[16] Sawada M, Suzumura A, Yamamoto H, Marunouchi T (1990) Activation and proliferation of the isolated microglia by colony stimulating factor-1 and possible involvement of protein kinase C. Brain Res. 509: 119–124. 10.1016/0006-8993(90)90317-5 2306629

[17] Zhang X, Goncalves R, Mosser DM (2008) The isolation and characterization of murine macrophages. Curr Protoc Immunol Chapter 14: Unit 14 11. 10.1002/0471142735.im1401s83 PMC2834554 19016445

[18] Morton GJ, Cummings DE, Baskin DG, Barsh GS, Schwartz MW (2006) Central nervous system control of food intake and body weight. Nature 443: 289–295. 16988703 10.1038/nature05026

[19] Romanatto T, Cesquini M, Amaral ME, Roman EA, Moraes JC, et al (2007) TNF-alpha acts in the hypothalamus inhibiting food intake and increasing the respiratory quotient–effects on leptin and insulin signaling pathways. Peptides 28: 1050–1058. 17459524 10.1016/j.peptides.2007.03.006

[20] Perry VH (2004) The influence of systemic inflammation on inflammation in the brain: implications for chronic neurodegenerative disease. Brain Behav Immun 18: 407–413. 15265532 10.1016/j.bbi.2004.01.004

[21] Thaler JP, Choi SJ, Schwartz MW, Wisse BE (2010) Hypothalamic inflammation and energy homeostasis: Resolving the paradox. Frontiers in Neuroendocrinology 31: 79–84. 19822168 10.1016/j.yfrne.2009.10.002

[22] Buchanan JB, Johnson RW (2007) Regulation of Food Intake by Inflammatory Cytokines in the Brain. Neuroendocrinology 86: 183–190. 17823502 10.1159/000108280

[23] Butera PC, Briffa CF, Whitaker EE (2004) Devazepide fails to reverse the inhibitory effect of interleukin-1beta on food intake in female rats. Physiol Behav 82: 777–783. 15451641 10.1016/j.physbeh.2004.06.018

[24] Plata-Salaman CR, Sonti G, Borkoski JP, Wilson CD, French-Mullen JMb (1996) Anorexia induced by chronic central administration of cytokines at estimated pathophysiological concentrations. Physiol Behav 60: 867–875. 9110949

[25] Zhang X, Zhang G, Zhang H, Karin M, Bai H, et al (2008) Hypothalamic IKKbeta/NF-kappaB and ER stress link overnutrition to energy imbalance and obesity. Cell 135: 61–73. 18854155 10.1016/j.cell.2008.07.043 PMC2586330

[26] Cloutier A, Guindi C, Larivée P, Dubois CM, Amrani A, et al (2009) Inflammatory Cytokine Production by Human Neutrophils Involves C/EBP Transcription Factors. The Journal of Immunology 182: 563–571. 19109189 10.4049/jimmunol.182.1.563

[27] Nathan C (2006) Neutrophils and immunity: challenges and opportunities. Nat Rev Immunol 6: 173–182. 16498448 10.1038/nri1785

[28] Chadzinska M, Kolaczkowska E, Scislowska-Czarnecka A, Van Rooijen N, Plytycz B (2004) Effects of macrophage depletion on peritoneal inflammation in swiss mice, edible frogs and goldfish. Folia Biol (Krakow) 52: 225–231. 19058564 10.3409/1734916044527557

[29] Gordy C, Pua H, Sempowski GD, He Y-W (2011) Regulation of steady-state neutrophil homeostasis by macrophages. Blood 117: 618–629. 20980680 10.1182/blood-2010-01-265959 PMC3031484

[30] Adachi K, Suzuki M, Sugimoto T, Uetsuka K, Nakamaya H, et al (2003) Effects of granulocyte colony-stimulating factor on the kinetics of inflammatory cells in the peripheral blood and pulmonary lesions during the development of bleomycin-induced lung injury in rats. Exp Toxicol Pathol 55: 21–32. 12940625 10.1078/0940-2993-00297

[31] Hierholzer C, Kelly E, Lyons V, Roedling E, Davies P, et al (1998) G-CSF instillation into rat lungs mediates neutrophil recruitment, pulmonary edema, and hypoxia. J Leukoc Biol 63: 169–174. 9468275 10.1002/jlb.63.2.169

[32] Moser B, Clark-Lewis I, Zwahlen R, Baggiolini M (1990) Neutrophil-activating properties of the melanoma growth-stimulatory activity. J Exp Med 171: 1797–1802. 2185333 10.1084/jem.171.5.1797 PMC2187876

[33] Dorshkind K (1990) Regulation of hemopoiesis by bone marrow stromal cells and their products. Annu Rev Immunol 8: 111–137. 2188660 10.1146/annurev.iy.08.040190.000551

[34] Rennick D, Yang G, Gemmell L, Lee F (1987) Control of hemopoiesis by a bone marrow stromal cell clone: lipopolysaccharide- and interleukin-1-inducible production of colony- stimulating factors. Blood 69: 682–691. 3492227

[35] Watari K, Ozawa K, Tajika K, Tojo A, Tani K, et al (1994) Production of human granulocyte colony stimulating factor by various kinds of stromal cells in vitro detected by enzyme immunoassay and in situ hybridization. Stem Cells 12: 416–423. 7524894 10.1002/stem.5530120409

[36] Odegaard JI, Ricardo-Gonzalez RR, Goforth MH, Morel CR, Subramanian V, et al (2007) Macrophage-specific PPARgamma controls alternative activation and improves insulin resistance. Nature 447: 1116–1120. 17515919 10.1038/nature05894 PMC2587297

[37] Odegaard JI, Ricardo-Gonzalez RR, Red Eagle A, Vats D, Morel CR, et al (2008) Alternative M2 activation of Kupffer cells by PPARdelta ameliorates obesity-induced insulin resistance. Cell Metab 7: 496–507. 18522831 10.1016/j.cmet.2008.04.003 PMC2587370

[38] Mantovani A, Cassatella MA, Costantini C, Jaillon S (2011) Neutrophils in the activation and regulation of innate and adaptive immunity. Nat Rev Immunol 11: 519–531. 21785456 10.1038/nri3024

[39] Wenzel P, Knorr M, Kossmann S, Stratmann J, Hausding M, et al (2011) Lysozyme M-positive monocytes mediate angiotensin II-induced arterial hypertension and vascular dysfunction. Circulation 124: 1370–1381. 21875910 10.1161/CIRCULATIONAHA.111.034470

